# GRWR Correlates with the Metabolism of Tacrolimus after Pediatric Living Donor Liver Transplantation According to Donor CYP3A5 Polymorphism

**DOI:** 10.1155/2022/7647754

**Published:** 2022-10-30

**Authors:** Ping Wan, Yuchen Hou, Bijun Qiu, Mingxuan Feng, Taihua Yang, Yi Luo, Lei Xia, Xiaosong Chen, Jianjun Zhang, Feng Xue, Qiang Xia

**Affiliations:** Department of Liver Surgery, Renji Hospital, School of Medicine, Shanghai Jiao Tong University, Shanghai, China

## Abstract

**Objectives:**

Tacrolimus is characterized by high pharmacokinetic variability in combination with a narrow therapeutic range. However, influence of donor CYP3A5 genotype and graft-to-recipient body weight ratio (GRWR) on tacrolimus' pharmacokinetics after pediatric living donor liver transplantation (LDLT) remains unclear.

**Methods:**

A total of 174 LDLT recipients (<6 y) were grouped according to donor CYP3A5 genotypes (nonexpressor (NEX) or expressor (EX)) and GRWR (<3.0% (SS, small-size) or ≥3.0% (LS, large-size)): SS/NEX (*n* = 40), SS/EX (*n* = 38), LS/NEX (*n* = 48), and LS/EX (*n* = 48). Pharmacokinetics of tacrolimus and clinical outcomes were analyzed.

**Results:**

The relationships between the concentration-dose ratio and donor CYP3A5 genotypes and graft size were examined 3, 7, 14, and 30 days after the transplantation. Tacrolimus C0 levels varied greatly among groups, although recipients started with the same initial dosage. LS/EX recipients had significantly lower C0 levels in comparison with those of other groups. The use of CYP3A5-EX-grafts and a greater GRWR both resulted in significantly higher TAC dose requirements and lower C/D ratios. However, the significance of GRWR no longer exists 3 months after transplantation. The multivariate generalized linear mixed model analysis showed that donor CYP3A5 genotypes (*F* = 11.876; *P* = 0.01) and GRWR (*F* = 4.631; *P* = 0.033) were independent impact factors for C/D ratios 3, 7, 14, and 30 days after transplantation. Donor CYP3A5-EX genotype was associated with significantly increasing risks of infectious complications and significantly lower Cylex ATP values. However, no significant difference was observed in acute rejections among 4 groups.

**Conclusions:**

Monitoring of C0 levels alone is not reliable to guide tacrolimus administration. Donor CYP3A5 and GRWR both significantly affect tacrolimus pharmacokinetics after pediatric LDLT. The use of Cylex ATP tests would be helpful to avoid overimmunosuppression.

## 1. Introduction

Grafts from living donors have currently become the first choice for pediatric liver transplantation (LT) in Asian countries because of the severe shortage of deceased donor organs in children. In experienced transplant centers, living donor liver transplantation (LDLT) in children has satisfactory short-term and long-term outcomes with the 5-year graft survival rate reaching more than 85% [1–4]. However, the long-term use of immunosuppressant brings a great challenge to posttransplantation medical management, which has to be balanced between the risks of overimmunosuppression, side effects, and rejections.

Tacrolimus (TAC) is the most common calcineurin inhibitor used for the prevention and treatment of rejection in patients undergoing solid organ transplantation. High TAC exposure leads to increased risks of infections, renal injury, or other toxic effects, while a low dosage of TAC may cause acute or chronic rejections, and even graft loss or death in severe cases. TAC has a narrow therapeutic range and a high interindividual variability in terms of pharmacokinetics [5–7]. Especially in pediatric recipients, a 10-fold variability in blood concentration and pharmacokinetic parameters at a fixed dose is frequently observed. Previous research found that the small graft size was associated with lower TAC metabolism in the early period after pediatric living donor liver transplantation [8]. Our previous study found that the cytochrome P450 (CYP) 3A5 genotype in both recipients and donors significantly affected TAC pharmacokinetics after LT in pediatric patients, of which donor CYP3A5 genotype was the major contributor accounting for 32.35% [9]. A recent research also found that donor CYP3A5 genotype and the age of pediatric liver recipients affect TAC pharmacokinetics [10]. CYP3A5 is found in small intestine as well as in the liver [11]. Therefore, based on our clinical investigation, we speculate that a larger liver graft carries more CYP3A5 oxidoreductase, which could accelerate the TAC's clearance. However, how CYP3A5 genotypes and graft-to-recipient weight ratio (GRWR) influence TAC concentration/dose (C/D) ratio in pediatric recipients remains unclear. In this study, we aim to explore the role of donor CYP3A5 and GRWR on TAC's pharmacokinetics after LDLT in pediatric cases.

## 2. Patients and Methods

### 2.1. Patients

Children who underwent LDLT before the age of 6 years between June 2009 and December 2014 were included in further patient screening. Exclusion criteria are shown as below: (1) retransplantation (0 case); (2) combined organ transplantation (0 case); (3) diagnosis with malignant tumor (0 case); (4) graft loss or death within 1 month after LT (9 cases); (5) use of cyclosporine-based regimen as the initial immunosuppression regimen (1 case); (6) data missing (17 cases). Finally, 174 children and their corresponding donors were included in our study and categorized into 4 groups by GRWR and donor CYP3A5 genotype: the SS (small-size)/NEX (nonexpressor) group had a GRWR < 3.0% and absence (^∗^3/^∗^3) of donor CYP3A5 (*n* = 40); the SS/EX (expressor) group had a GRWR < 3.0% and presence (^∗^1/^∗^1 or ^∗^1/^∗^3) of donor CYP3A5 (*n* = 38); the LS (large-size)/NEX group had a GRWR ≥ 3.0% and absence of donor CYP3A5 (*n* = 48); and the SS/EX group had a GRWR ≥ 3.0% and presence of donor CYP3A5 (*n* = 48). The baseline characteristics and TAC's pharmacokinetic parameters were analyzed and compared between the 4 groups. This study was approved by the institutional review board of Renji Hospital, and written informed consents were obtained from all parents of recipients.

### 2.2. Immunosuppressive Protocol

A dual drug regimen of TAC combined with methylprednisolone (4 mg/kg/day) was used as the initial immunosuppressive regimen. TAC was started on day 1 after LT with an initial dosage at 0.1-0.15 mg/kg/day, and the target trough level (termed C0 level) was 8-12 ng/ml during the first month after LT, 7-10 ng/ml from the 2nd to 6th month, 5-8 ng/ml from the 7th to 12th month, and around 5 ng/ml thereafter. If TAC could not reach the target C0 level despite a twice increase of dosage, patients would use a triple-drug regimen with mycophenolate mofetil added or switch to a cyclosporine-based regimen. The use of methylprednisolone was started at 10 mg/kg during LT and at 4 mg/kg on day 1 after LT. The dose of methylprednisolone was gradually tapered by 4 to 8 mg per day and maintained with prednisone 2.5 to 5 mg daily taken orally, which was withdrawn within 3 months after LT [12].

### 2.3. TAC C0 Monitoring and C/D Ratio Assessment

One milliliter of ethylenediamine tetraacetic acid-treated venous whole blood was collected 12 hours after previous TAC administration. Blood TAC C0 level was tested using the microparticulate enzyme immunoassay (Abbott Co., Ltd, Tokyo, Japan). TAC's weight-adjusted doses and C0 levels were recorded after each blood test, and C/D ratios were calculated using the C0 levels (ng/ml) divided by the weight-adjusted dose (mg/kg/d), which was used as an alternative method for estimation of TAC clearance [9, 13, 14]. TAC weight-adjusted doses and C/D ratios at 3 days, 7 days, 14 days, 1 month, 3 months, 6 months, and 12 months after LT were included as primary endpoints in this study. If the TAC C0 level was not available at a given time point, the data were excluded from the analysis.

### 2.4. Genotyping of CYP3A5

The single nucleotide polymorphism of CYP3A5 at position 6986*A* > *G* (the ^∗^3 or ^∗^1 allele, rs776746) was tested for the 174 children and their corresponding donors. We extracted the genomic DNA from leukocytes using a QIAamp Blood Kit (Qiagen, Hilden, Germany). The PCR-based sequencing process and primer information were performed as described previously [11]. The forward primer was 5′-ACTGCCCTTGCAGCATTTA-3′, and the reverse primer was 5′-CCAGGAAGCCAGACTTTGA-3′. Briefly, the following program was utilized: 95°C for 10 min, 94°C for 30 s, 55°C for 30 s, 72°C for 60 s for total 40 cycles, and finally 72°C for 7 min. Afterwards, products were purified with QIAquick PCR purification kit (Qiagen, Hilden, Germany), and the fragments were run on an ABI 3730XL Genetic Analyzer.

### 2.5. Immune Function Assay (Cylex ATP)

CD4 + T-lymphocyte functional assay (ImmuKnow, Catalogue no. 4400, Cylex Inc., Columbia, MD) was performed for assessment of cellular immunity functions according to our previous study [15]. In brief, 250 ul whole blood was added to 750 ul sample diluent to obtain 1000 ul solution, and 100 ul samples were incubated overnight (15-18 h) with phytohemagglutinin stimulation in wells of a 96-well microtiter plate at 37°C in a 5% CO2 incubator. CD4 + T-cells were selected by magnetic particles coated with antihuman CD4 monoclonal antibodies within the microwells and then lysed to release intracellular ATP. After the addition of luciferin/luciferase reagents into each wells of the plate, ATP values were measured using a luminometer to reflecting functional activity of lymphocytes. ATP values at 21 days after LT were analyzed in this study. We used pediatric ImmuKnow assay zones proposed by Hooper et al. [16] for evaluation of over- or under-immunosuppression, and strong, moderate, and low immune function corresponded to >395 ng/ml, 175-395 ng/ml, and <175 ng/ml, respectively.

### 2.6. Statistical Analysis

SPSS 19.0 software (SPSS Inc. Chicago, IL, United States) was used for statistical analysis. *P* values <0.05 indicated statistically significant. All measurement data were tested for normality using the Shapiro-Wilk method. The independent-samples *t*-test analysis was used for variables with normal distribution, while the nonparametric statistical analysis (Mann–Whitney test) was applied to abnormal-distribution variables. Categorical data were analyzed using *χ*2 test. The generalized linear mixed model (GLMM) was used to analyze each factor that might have influenced TAC C/D ratios, and any variables identified as statistically significant in the univariate GLMM analysis were included in the multivariate GLMM analysis to obtain independent factors affecting TAC C/D ratios. Logistic analysis was used to study the correlations between CYP3A5 genotype and infection, rejection status, respectively, then the relationship between CYP3A5 genotype and infection, rejection status, respectively. The rejection-free survival rate was estimated using Kaplan-Meier method (the time interval between LT and the first episode of acute rejection), and differences between groups were compared using the log-rank test.

## 3. Results

### 3.1. Patient and Donor Characteristics

Detail frequencies of CYP3A5 genotypes in donors and recipients are shown in Table 1. The CYP3A5 NEX and EX phenotypes were found in 88 (50.6%) and 86 (49.4%) donors, respectively, and were observed in 96 (55.2%) and 78 (44.8%) recipients, respectively. Allele frequencies in donors were 104 (29.9%) for ^∗^1 and 244 (70.1%) for ^∗^3, and those in recipients were 85 (24.4%) for ^∗^1 and 263 (75.6%) for ^∗^3, respectively.  Table 2 shows recipient characteristics of the 4 groups. There were 86 (49.4%) boys and 88 (50.6%) girls, with a median age of 8.7 months (range from 4.8 to 71.5 months). The median body weight and height were 7.7 kg (range from 5.1-28.0 kg) and 66 cm (range from 56-120 cm), respectively. Pediatric end-stage liver disease (PELD) scores of <0, 0-9, 10-19, and ≥20 were seen in 15 (8.6%), 23 (13.2%), 77 (44.3%), and 59 (33.9%) patients, respectively. Sixty-four (36.8%) and 110 (63.2%) patients underwent LDLT in the first stage (2009-2012) and in the second stage (2013-2014), respectively.  Table 3 shows characteristics of living donors in 4 groups. One hundred and sixty-six (95.4%) donors were parents, and 8 (4.6%) donors were grandparents. They included 74 (42.5%) men and 100 (57.5%) women, with a median age of 30 years (range from 20 to 56 years). The median body weight and height were 58 kg (range from 39-90 kg) and 165 cm (range from 148-184 cm), respectively. Moreover, patients receiving donor livers of NEX and EX phenotypes had median GRWR of 3.1% (range from 1.3 to 5.8%) and 3.0% (range from 1.2 to 5.7%), respectively.

### 3.2. TAC C0 Levels

**Figure 1:**
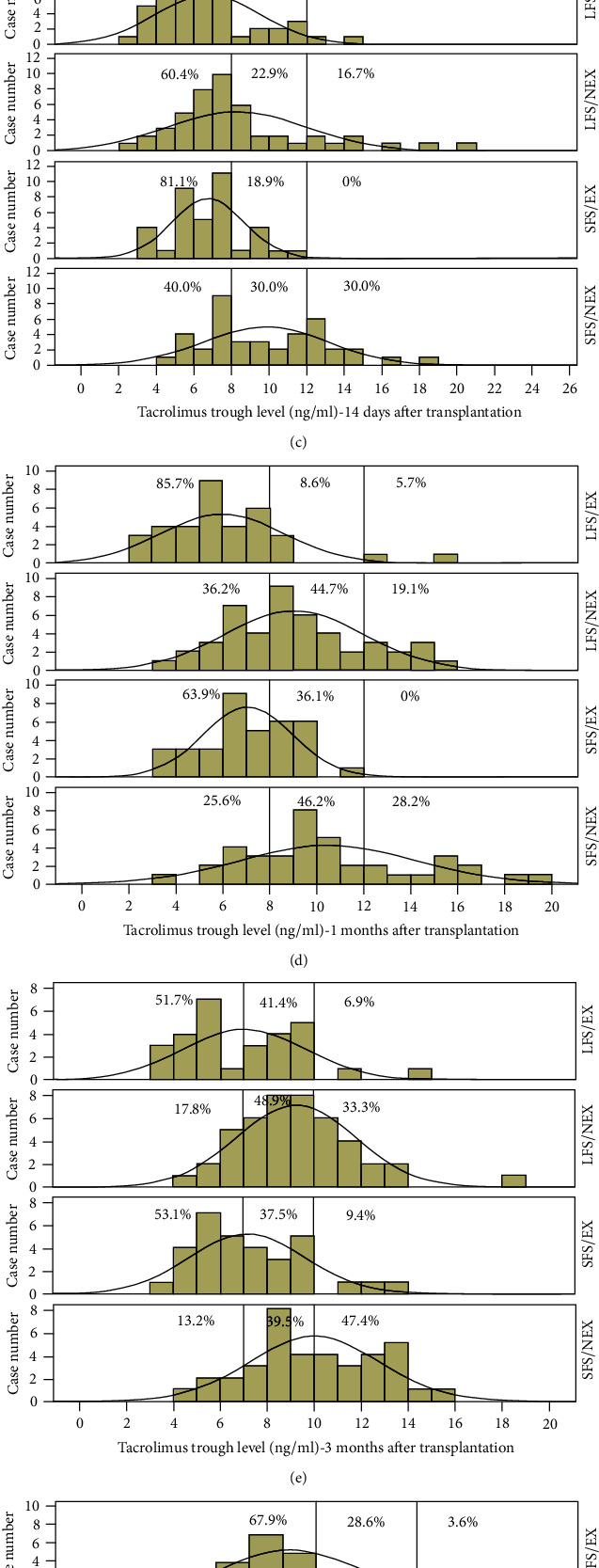
Comparison of tacrolimus trough level distributions among 4 groups at different time points. (a) 3 days; (b) 7 days; (c) 14 days; (d) 1 month; (e) 3 months; (f) 6 months. (g) 12 months. Pecentages indicate proportions of patients in different groups whose trough level is under the lower limit, within the target range or beyond the upper limit. The area between two vertical lines denotes the target range. The curves are normal distribution curves.

TAC dosages were adjusted according to target C0 levels at different time point after LT. However, we found that most C0 levels were outside the target ranges, and proportions of C0 levels within the target ranges were only 28.9%, 42.0%, 22.4%, 35.0%, 42.4%, 32.3%, and 30.4% at 3 days, 7 days, 14 days, 1 month, 3 months, 6 months, and 12 months after LT, respectively. Moreover, C0 levels varied greatly among SS/NEX, SS/EX, LS/NEX, and LS/EX groups, even though recipients started with the same initial dosage. Figures 1(a)–1(g) show TAC C0 level distributions in the 4 groups at 3 days, 7 days, 14 days, 1 month, 3 months, 6 months, and 12 months after LT, respectively. At all-time points, the SS/NEX group tended to have the smallest proportions of C0 levels under the lower limit of target ranges and the largest proportions of C0 levels beyond the upper limit of target ranges, which indicated that the highest C0 levels were most likely to appear in NEX-graft recipients with a small GRWR. On the contrary, the LS/EX group usually had the largest proportions of C0 levels under the lower limit of target ranges and the smallest proportions of C0 levels beyond the upper limit of target ranges, indicating a large GRWR combined with an EX-type graft brought about the lowest C0 levels. Twenty-seven children (15.5%) converted into a cyclosporine-based regimen at a median duration of 22 days (range from 8 to 270 days) after LT, of which 18 cases (66.7%) were from the LS/EX group. Mycophenolate mofetil was added at a median duration of 20 days (range from 4 to 232 days) after LT in 91 children (52.3%) (Table 4).

### 3.3. TAC Dose Requirement and C/D Ratio

**Figure 2:**
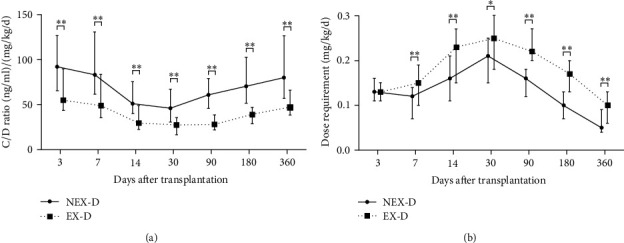
Comparison of tacrolimus dose requirements (a) and C/D ratio (b) between NEX- and EX-grafts recipients. The whiskers mark quartiles; ^∗^ indicates *P* < 0.05 between 2 groups; ^∗∗^ indicates *P* < 0.01 between 2 groups.

**Figure 3:**
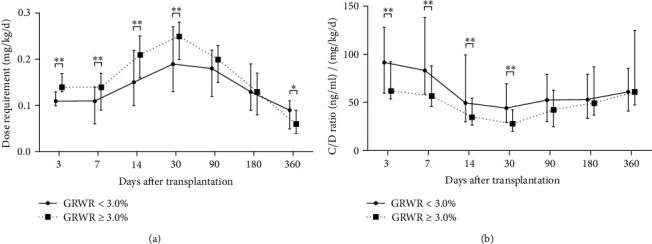
Comparison of tacrolimus dose requirements (a) and C/D ratio (b) between recipients with GRWR < 3.0% or GRWR ≥ 3.0%. The whiskers mark quartiles; ^∗^ indicates *P* < 0.05 between 2 groups; ^∗∗^ indicates *P* < 0.01 between 2 groups.

Compared with NEX-graft recipients, EX-graft recipients had a significant higher TAC dose requirement as well as a significant lower C/D ratio within 12 months after LT (Figure 2). Furthermore, a GRWR ≥ 3.0% was also associated with a significant higher TAC dose requirement and a significant lower C/D ratio from 3 days to 1 month after LT, but the significance lost between 3 to 12 months (Figure 3). The TAC dose requirement and C/D ratios of SS/NEX, SS/EX, LS/NEX, and LS/NEX groups at different time points were shown in Tables 5 and 6, respectively. In contrast to the SS/NEX group, a significant higher dose requirement and a significant lower C/D ratio were observed in the SS/EX group and LS/EX group from 3 days to 12 months after LT, and the significance was also shown in the LS/NEX group within 3 months after LT. Thus, the use of EX-type graft and a larger GRWR facilitated TAC clearance and caused an increase in dosage, but the impact of GRWR vanished 3 months after LT. The difference in dosage requirement between SS/NEX and LS/EX groups at 12th month failed to reach statistical significance probably because of more than 1/3 of recipients having converted into a cyclosporine-based regimen in the LS/EX group.

### 3.4. Uni- and Multivariate Analyses for TAC C/D Ratio

A total of 18 variables that might have affected TAC C/D ratios were subject to univariate GLMM analysis one by one using C/D ratios 3, 7, 14, and 30 days after LT as outcome variables (repeated measures), including recipient age (<12 months, 12-36 months, or ≥36 months), recipient gender, recipient weight (<7 kg, 7-10 kg, or ≥10 kg), recipient height (<70 cm or ≥70 cm), recipients' ABO blood group, ABO compatibility (identical, compatible, or incompatible), PELD score (<0, 0-9, 10-19, or ≥20), primary diagnosis (cholestatic liver disease or other), previous Kasai procedure (with or without), graft weight (<250 g or ≥250 g), GRWR (<3.0% or ≥3.0%), recipients' CYP3A5 genotype (EX or NEX), donor CYP3A5 genotype (EX or NEX), donor age (<40 years or ≥40 years), donor gender, donor body mass index (BMI) (<18.5, 18.5-24.0, or ≥24.0), donor ABO blood group, and donor-to-recipient relationship (father, mother, grandfather, and grandmother). We found that 8 variables had significant impacts, including recipient age, recipient weight, recipient height, PELD score, GRWR, recipient CYP3A5, donor CYP3A5, and donor ABO blood group. Finally, the multivariate GLMM analysis showed that donor CYP3A5 (*F* = 11.876, *P* = 0.01) and GRWR (*F* = 4.631, *P* = 0.033) were independent impact factors for C/D ratios 3, 7, 14, and 30 days after LT (Table 7). Similarly, the 18 aforementioned variables were subjected to uni- and multivariate GLMM analyses again using C/D ratios 3, 6, and 12 months after LT as outcome variables (repeated measures), which revealed that donor CYP3A5 (*F* = 20.998, *P* < 0.001), recipient CYP3A5 (*F* = 5.363, *P* = 0.022), and the ABO compatibility (*F* = 4.159, *P* = 0.018) were independent impact factors for C/D ratios 3, 6, and 12 months after LT (Table 8).

### 3.5. Posttransplant Infections and Acute Rejections

**Figure 4:**
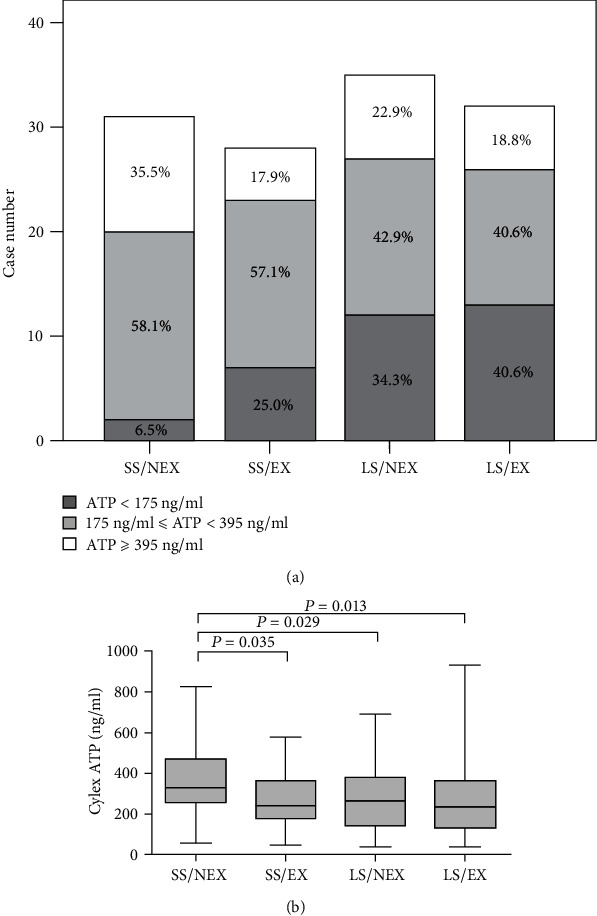
Comparison of Cylex ATP values among different groups. (a) Box-plots of Cylex ATP values (the boxes mark median values and quartiles, and the whiskers mark maximum and minimum values). (b) Proportions of patients with strong, moderate, and low immune functions. Low immune response: ATP < 175 ng/ml; moderate immune response: 175 ng/ml ≤ ATP < 395 ng/ml; strong immune response: ATP ≥ 395 ng/ml.

A total of 90 (51.7%) children experienced one or more episodes of infections within 3 months after LT, in which incidences of bacterial infection, fungal infection, Epstein-Barr virus infection, cytomegalovirus infection, and other infections were 35.1%, 5.2%, 16.1%, 16.1%, and 2.3%, respectively. Posttransplant infections were classified according to 2012 International Guidelines for Management of Severe Sepsis and Septic Shock [17]. In comparison with the SS/NEX group, significantly higher risks of sepsis were observed in both of the LS/EX (25.0% vs. 52.1%, *P* = 0.010) and SS/EX groups (25.0% vs. 50.0%, *P* = 0.022), but the LS/NEX group merely showed a slight trend in the increasing risk of sepsis (25.0% vs. 39.6%, *P* = 0.147) (Table 9). Namely, recipient receiving an EX-graft suffered from much higher risks of sepsis within 3 months after LT, but the GRWR had a slight influence on posttransplant infections. Cylex ATP levels, which are positively correlated with the cellular immunity activity, provided a useful approach in monitoring posttransplant infections [13–15]. As shown in Figure 4(a), the SS/EX group (median: 241.0 ng/ml; range from 45.1 to 577.7 ng/ml; *P* = 0.0035), the LS/NEX group (median: 266.0 ng/ml; range from 40.0 to 690.4 ng/ml; *P* = 0.0029), and the LS/EX group (median: 236.5 ng/ml; range from 40.0 to 935.0 ng/ml; *P* = 0.0013) all presented significantly lower Cylex ATP levels compared with the SS/NEX group (median: 329.1 ng/ml; range from 57.2 to 827.1 ng/ml). Meanwhile, we found that the LS/EX group had the highest proportion of children with low immune functions (ATP value < 175 ng/ml), due to an increasing use of TAC doses to obtain satisfactory C0 levels (Figure 4(b)). What is more, patients with donor CYP3A5-expressed (adjusted OR 2.04, CI 1.06-3.92, and *P* = 0.032) were likely to have bacterial infection after LT (Table 10). The results were adjusted by the conversion (to cyclosporine) or expansion (by adding MMF) of antirejection therapy.

**Figure 5:**
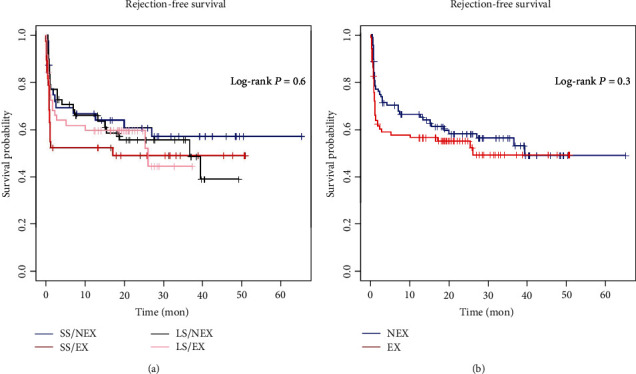
(a) Comparison of posttransplant rejection-free survival rates among different groups (*P* = 0.6). (b) Comparison of posttransplant rejection-free survival rates between patients with donor CYP3A5 expressed or nonexpressed (*P* = 0.3).

Rejection-free survival rates in SS/NEX, SS/EX, LS/NEX, and LS/EX groups were 67.0%, 52.6%, 66.1%, and 59.8% at 1 year, 57.4%, 49.3%, 55.9%, and 44.9% at 3 years, and 55.0%, 44.7%, 54.1%, and 43.8% at 5 years, respectively. No significant difference was observed in rejection-free survival rate between these groups (Figure 5(a), *P* = 0.6). However, CYP3A5-expressed patients prone to have shorter rejection-free survival time (Figure 5(b), *P* = 0.3).

## 4. Discussion

TAC's therapeutic use is complicated by its pharmacokinetic variability. The variable pharmacokinetic property of immunosuppressants may have a significant adverse effect on long-term outcomes after solid organ transplantation, secondary to an increased risk of acute and chronic rejections and drug toxicity. Thus, periodical monitoring of blood TAC C0 levels is recommended to judge whether TAC has reached the therapeutic window and detects possible rejections or overimmunosuppression. TAC's pharmacokinetic in transplantation is affected by various factors such as the patient age, ethnic groups, genetic factor, transplant organ type, coadministration with food or medication, gastrointestinal functions, and small intestinal p-glycoprotein [18–24]. In pediatric patients, TAC's oral absorption is inconstant, together with their reduced bioavailability and increased clearance in comparison with adult patients, resulting in a higher oral dose required to achieve target blood concentrations [20]. Moreover, small children may be expected to show more significant heterogeneity in TAC pharmacokinetic profiles, which makes it very difficult to administer an appropriate initial dose for each individual [9]. These features lead to higher risks of organ rejections, toxicity, and infections in pediatric patients.

TAC is metabolized by CYP3A enzymes into demethylated and hydroxylated metabolites, which catalyses metabolic reactions with extensive effects on the biological activities of drugs, pretoxic chemicals, and endogenous substances in human liver and small intestine [25]. CYP3A5 accounts for more than half of the total CYP3A content in people who polymorphically express CYP3A5. People with a CYP3A5^∗^1 allele (the wild type) have a high amount of CYP3A5 enzymes in the liver and small intestine. Ethnicity-dependent pharmacokinetic differences were reported in expression of CYP3A5, with African people (60%) characterized by more active CYP3A5 enzymes than white people (33%) [21, 26]. Our team has reported the expression rate of CYP3A5^∗^1 and ^∗^3 alleles in Chinese population [9], and data were verified in the present study that expression rate of CYP3A5 ^∗^1 in Chinese people was approximately 24-30%, and those who carried the ^∗^1 allele consisted of at least 40% of all cases. Based on our previous results, CYP3A5 genotypes in both donors and recipients had consistent influences on pharmacokinetic profile of TAC with contributions of 32.35% and 6.0% to the attributable variance in the C/D ratio, respectively [9]. It is understandable that the amount of CYP3A5 enzymes in liver grafts might be correlated with the graft size. In this study, we first verified an independently significant impact of GRWR on TAC C/D ratio in children undergoing LDLT using multivariate GLMM analyses. However, the liver graft size grows fast as regeneration of the liver until 3 months after LDLT, but the GRWR was negatively correlated with the regeneration rate [27, 28], which explains why GRWR on C/D ratio lost its significance between 3 to 12 months.

Interestingly, weight-adjusted doses of TAC were often much higher in infantile recipients than in older children regardless of CYP3A5 genotypes, indicating a decrease of TAC clearance corresponding to children's growth. Small children usually require approximately twice as much as weight-adjusted doses of TAC in adults to achieve the same C0 levels. However, younger children often tend to have a higher GRWR in LDLT using left lateral segment. The relationship between recipient age and GRWR probably plays an important role in pharmacokinetic variability of TAC over age, and this may explain why recipient age or weight are not independent impact factors on the TAC C/D ratio. In this study, results were much more explicit and reliable using multivariate GLMM analyses with C/D ratios in multiple levels (different time points after LT), which minimized the possible interlevel selection bias in single-level regression model. The recipient CYP3A5 genotype determines the amount of CYP3A5 protein in the small intestine, where TAC is absorbed. TAC is highly lipophilic and easily spreads into intestinal epithelial cells after oral administration. CYP3A5 enzymes in the small intestine reduce the oral bioavailability of TAC [29–31]. Consequently, recipient CYP3A5 genetypes show a tendency towards statistical significance regarding its impact on C/D ratio within 1 month after LT. It is noteworthy that 2 of the 5 ABO-incompatible LT recipients (40%) had switched to a cyclosporine-based regimen due to unsatisfactory TAC C0 levels, so the significance of ABO compatibility in the multivariate GLMM analysis might be caused by the limited number of cases.

Our data show children with an EX-graft suffered from higher risks of infections after LT than those with a NEX-graft even if they have lower C0 levels of TAC, suggesting that the TAC C0 level is not a reliable index to truly reflect patients' situation of immunosuppression. However, this is probably related to the increasing doses of TAC to reach the target C0 levels in this group of patients. Alak and Moy [32] revealed that only 54.4% of the immunosuppression was ascribed to TAC after transplantation. TAC is metabolized by CYP3A enzymes into at least 4 major metabolites (M-I, M-II, M-III, and M-IV) and at least 9 total metabolites, which might have dissimilar immunosuppressant activities. The most common metabolite is the 13-0-demethyl FK506 (M-I), and other metabolites include 0-demethylated at the 31 and 15 positions (M-II and M-III, respectively) and hydroxylated at the 12 position (M-IV) [33–36]. On the other hand, this study suggests that risks of acute rejections are not influenced by donor CYP3A5 genotype or GRWR. To further evaluate the immune functions status of recipients, we compared Cylex ATP values using CD4 + T-lymphocyte functional assay after LT. As expected, the LS/EX group who had the highest risk of infectious complications was associated with highest proportion of patients with the ATP value < 175 ng/ml, but the GRWR had less impact on incidence of infectious complications than donor CYP3A5 genotypes. Nonetheless, we recommend that donor CYP3A5 genotypes and the GRWR should be taken into consideration during administration of TAC after pediatric LDLT.

There are several underlying limitations in our study. First, data in this study were retrospectively reviewed from the pediatric LT database, and the sample size was subject to the nature of a single-center study. Second, disproportionate percentages of children who switched from TAC to cyclosporine across the 4 groups might cause selection bias in multivariate GLMM analyses. Third, amounts of TAC metabolites in recipients and CYP3A5 enzyme activities in donor livers were not measured in this study, and how TAC metabolites affect immune functions after pediatric LDLT still needs to be further clarified in a prospective study.

In conclusion, donor CYP3A5-EX-type and a greater GRWR are both associated with significantly higher TAC dose requirements and lower C/D ratios after pediatric LDLT. However, significance of GRWR vanished after 3 months posttransplant. High doses of TAC in recipients with EX-grafts would result in increasing risks of infectious complications. Thus, monitoring of immune responses using Cylex ATP levels would be helpful to establish individualized immunosuppressant regimens.

## Figures and Tables

**Table 1:** CYP3A5 genotype distribution in 174 recipients and their donors.

Donor CYP3A5 genotype	Recipient CYP3A5 genotype			
	^∗^1/^∗^1 (EX)	^∗^1/^∗^3 (EX)	^∗^3/^∗^3 (NEX)	Overall
^∗^1/^∗^1 (EX)	3	12	3	18 (10.3)
^∗^1/^∗^3 (EX)	3	43	22	68 (39.1)
^∗^3/^∗^3 (NEX)	1	16	71	88 (50.6)
Overall	7 (4.0)	71 (40.8)	96 (55.2)	174 (100.0)

Note: Data are expressed as number (%).

**Table 2:** Baseline characteristics of recipients.

Variable	SS/NEX group (*n* = 40)	SS/EX group (*n* = 38)	LS/NEX group (*n* = 48)	LS/EX group (*n* = 48)
Age (month)	12.2 (5.7-71.5)	10.9 (5.7-63.4)	7.6 (5.1-22.2)	8.1 (4.8-53.6)
Gender				
Boy	24 (60.0)	19 (50.0)	20 (41.7)	23 (47.9)
Girl	16 (40.0)	19 (50.0)	28 (58.3)	25 (52.1)
Weight (kg)	9.3 (5.9-28.0)	9.0 (6.0-18.0)	6.9 (5.1-12.0)	7.0 (5.3-10.5)
Height (cm)	70 (60-120)	70.5 (58-108)	65 (56-76)	65 (57-88)
PELD score	13 (-9-28)	16 (-10-29)	17 (-2-36)	19 (-9-37)
ABO blood group				
A	16 (40.0)	8 (21.1)	11 (22.9)	10 (20.8)
B	9 (22.5)	12 (31.6)	13 (27.1)	13 (27.1)
O	9 (22.5)	16 (42.1)	19 (39.6)	19 (39.6)
AB	6 (15.0)	2 (5.3)	5 (10.4)	6 (12.5)
ABO compatibility				
Identical	30 (75.0)	32 (84.2)	39 (81.3)	35 (72.9)
Compatible	9 (22.5)	4 (10.5)	8 (16.7)	12 (25.0)
Incompatible	1 (2.5)	2 (5.3)	1 (2.1)	1 (2.1)
Etiology				
Biliary atresia	31 (77.5)	35 (92.1)	47 (97.9)	47 (97.9)
Cholestatic liver cirrhosis	0	1 (2.6)	0	0
PFIC	2 (5.0)	0	0	1 (2.1)
Cryptogenic liver cirrhosis	0	1 (2.6)	0	0
Tyrosinemia	4 (10.0)	0	0	0
Abernethy syndrome	1 (2.5)	0	0	0
Glycogen storage disease	1 (2.5)	0	0	0
Caroli disease	0	0	1 (2.1)	0
Alagille syndrome	0	1 (2.6)	0	0
Niemann-Pick disease	1 (2.5)	0	0	0
Kasai procedure				
Yes	16 (40.0)	19 (50.0)	21 (43.8)	14 (29.2)
No	24 (60.0)	19(50.0)	27 (56.3)	34 (70.8)
Transplant stage				
2009-2012	18 (45.0)	20 (52.6)	17 (35.4)	9 (18.8)
2013-2014	22 (55.0)	18 (47.4)	31 (64.6)	39 (81.3)

Note: Data are expressed as number (%) or median (range). GRWR: graft-to-recipient body weight ratio; PELD: pediatric end-stage liver disease; PFIC: progressive familial intrahepatic cholestasis.

**Table 3:** Donor characteristics.

Variable	SS/NEX group (*n* = 40)	SS/EX group (*n* = 38)	LS/NEX group (*n* = 48)	LS/EX group (*n* = 48)
Age (year)	30 (20-54)	30 (21-56)	29 (22-51)	31 (21-50)
Gender				
(i) Male	19 (47.5)	18 (47.4)	22 (45.8)	15 (31.3)
(ii) Female	21 (52.5)	20 (52.6)	26 (54.2)	33 (68.8)
Weight (kg)	57 (39-90)	56 (39-76)	60 (42-81)	60 (40-85)
Height (cm)	163 (150-183)	165 (152-179)	167 (150-180)	164 (148-184)
BMI (kg/m^2^)	21.1 (16.7-29.1)	20.6 (16.9-26.3)	21.6 (17.5-28.0)	22.9 (16.0-26.6)
D/R relationship				
Parent	39 (97.5)	36 (94.7)	45 (93.7)	46 (95.8)
Grandparent	1 (2.5)	2 (5.3)	3 (6.3)	2 (4.2)
Graft weight (g)	230 (140-420)	220 (130-345)	263 (190-370)	258 (180-395)
GRWR (%)	2.5 (1.3-3.0)	2.3 (1.2-3.0)	3.8 (3.0-5.8)	3.5 (3.0-5.7)
Graft type				
LLS	33 (82.5)	33 (86.8)	46 (95.8)	41 (85.4)
Reduced LLS	1 (2.5)	1 (2.6)	2 (4.2)	6 (12.5)
Left lobe without MHV	1 (2.5)	1 (2.6)	0	0
Left lobe with MHV	5 (12.5)	3 (7.9)	0	0
Right lobe without MHV	0	0	0	1 (2.1)

Note: Data are expressed as number (%) or median (range). BMI: body mass index; D/R donor/recipient; GRWR: graft-to-recipient weight ratio; LLS: left lateral segment; MHV: middle hepatic vein.

**Table 4:** Change of immunosuppressive regimens.

	SS/NEX group (*n* = 40)	SS/EX group (*n* = 38)	LS/NEX group (*n* = 48)	LS/EX group (*n* = 48)
FK506-to-CSA conversion				
Case number (%)	2 (5.0)	6 (15.8)	1 (2.1)	18 (37.5)
Starting days after LT	143 (15-270)	32 (8-122)	14	22 (10-75)
Additional use of MMF				
Case number	17 (42.5)	30 (78.9)	16 (33.3)	29 (60.4)
Starting days after LT	20 (5-57)	22 (6-140)	20 (4-84)	18 (5-232)

Note: Data are expressed as number (%) or median (range). CSA: cyclosporine; MMF: mycophenolate mofetil; LT: liver transplantation.

**Table 5:** Comparison of TAC dose requirement among the 4 groups.

Posttransplant time	TAC dose requirement (mg/kg/d)			
	SS/NEX group (*n* = 40)	SS/EX group (*n* = 38)	LS/NEX group (*n* = 48)	LS/EX group (*n* = 48)
3 days	0.11 (0.09-0.13)	0.12 (0.11-0.16)^∗^	0.14 (0.13-0.17)^∗∗^	0.14 (0.12-0.15)^∗∗^
7 days	0.10 (0.06-0.13)	0.12 (0.08-0.16)^∗∗^	0.13 (0.08-0.17)^∗∗^	0.18 (0.13-0.20)^∗∗^
14 days	0.13 (0.08-0.17)	0.21 (0.12-0.26)^∗∗^	0.17 (0.14-0.24)^∗∗^	0.25 (0.18-0.28)^∗∗^
1 month	0.18 (0.10-0.23)	0.22 (0.17-0.32)^∗∗^	0.23 (0.19-0.27)^∗∗^	0.25 (0.21-0.32)^∗∗^
3 months	0.14 (0.11-0.17)	0.23 (0.18-0.28)^∗∗^	0.18 (0.13-0.22)^∗∗^	0.22 (0.20-0.24)^∗∗^
6 months	0.10 (0.07-0.12)	0.18 (0.13-0.23)^∗∗^	0.11 (0.07-0.14)	0.16 (0.10-0.18)^∗∗^
12 months	0.07 (0.04-0.09)	0.11 (0.08-0.16)^∗∗^	0.05 (0.04-0.09)	0.08 (0.04-0.12)

Note: Data are expressed as median (lower quartile-upper qurtile). ^∗^ indicates *P* < 0.05 compared with the SS/NEX group; ^∗∗^ indicates *P* < 0.01 compared with the SS/NEX group.

**Table 6:** Comparison of TAC C/D ratio among the 4 groups.

Posttransplant time	TAC C/D ratio (ng/ml)/(mg/kg/d)			
	SS/NEX group (*n* = 40)	SS/EX group (*n* = 38)	LS/NEX group (*n* = 48)	LS/EX group (*n* = 48)
3 days	113.7 (99.2-150.0)	62.5 (43.4-100.3)^∗∗^	74.4 (59.9-100.0)^∗∗^	51.5 (43.2-82.2)^∗∗^
7 days	104.9 (70.2-192.0)	75.3 (40.7-117.4)^∗∗^	70.4 (57.2-102.4)^∗∗^	41.1 (31.1-76.8)^∗∗^
14 days	69.1 (51.8-132.4)	31.6 (24.8-59.1)^∗∗^	42.4 (33.6-58.0)^∗∗^	28.1 (20.3-46.4)^∗∗^
1 month	59.7 (33.8-89.2)	31.7 (18.9-49.0)^∗∗^	34.9 (25.6-50.8)^∗∗^	20.5 (16.2-28.7)^∗∗^
3 months	71.1 (53.4-91.8)	30.3 (20.6-47.5)^∗∗^	53.8 (42.5-68.7)^∗∗^	27.3 (21.9-37.1)^∗∗^
6 months	75.6 (60.8-103.7)	34.0 (27.4-47.8)^∗∗^	69.0 (46.0-95.1)	41.3 (29.2-47.3)^∗∗^
12 months	80.0 (57.3-92.8)	42.0 (29.6-63.5)^∗∗^	79.1 (56.0-136.0)	49.5 (41.0-85.7)^∗∗^

Note: Data are expressed as median (lower quartile-upper qurtile). ^∗^ indicates *P* < 0.05 compared with the SS/NEX group; ^∗∗^ indicates *P* < 0.01 compared with the SS/NEX group.

**Table 7:** Uni- and multivariate analyses of variables that significantly affected TAC C/D ratios 3, 7, 14, and 30 days after LT.

Significant variables	Univariate analysis		Multivariate analysis	
	*F* value	*P* value	*F* value	*P* value
Recipient age	15.907	<0.001	/	/
Recipient weight	11.064	<0.001	/	/
Recipient height	10.150	0.002	/	/
PELD score	6.096	0.001	/	/
GRWR	17.105	<0.001	4.631	0.033
Donor CYP3A5	12.311	0.001	11.876	0.01
Recipient CYP3A5	21.781	<0.001	/	/
Donor ABO blood group	2.874	0.038	/	/

Note: GRWR: graft-to-recipient weight ratio; PELD: pediatric end-stage liver disease; TAC: tacrolimus; C/D: concentration-dose; LT: liver transplantation.

**Table 8:** Uni- and multivariate analyses of variables that significantly affected TAC C/D ratios 3, 6, and 12 months after LT.

Significant variables	Univariate analysis		Multivariate analysis	
	*F* value	*P* value	*F* value	*P* value
ABO compability	4.168	0.018	4.159	0.018
Donor CYP3A5	40.665	<0.001	20.998	<0.001
Recipient CYP3A5	28.398	<0.001	5.363	0.022

Note: TAC: tacrolimus; C/D: concentration-dose; LT: liver transplantation.

**Table 9:** Comparison of infectious complications within 3 months among the 4 groups.

Infection	SS/NEX group (*n* = 40)	SS/EX group (*n* = 38)	LS/NEX group (*n* = 48)	LS/EX group (n =48)
No infection	24 (60.0)	17 (44.7)	25 (52.1)	19 (39.5)
Local infection	6 (15.0)	2 (5.3)	4 (8.3)	4 (8.3)
Sepsis	10 (25.0)	19 (50.0)^∗^	19 (39.6)	25 (52.1)^∗^
No organ dysfunction	10	15	14	23
With organ dysfunction	0	4	5	2

Note: ^∗^ indicates *P* < 0.05 compared with the SS/NEX group.

**Table 10:** Associations between CYP3A5 genotype, GRWR, and rejection status and infection.

	Rejection status				Infection			
	Unadjusted OR (95% CI)	*P* value	Adjusted OR (95% CI)	*P* value	Unadjusted OR (95% CI)	*P* value	Adjusted OR (95% CI)	*P* value
Recipient CYP3A5 genotypes								
Nonexpressor	Ref.	Ref.	Ref.	Ref.	Ref.	Ref.	Ref.	Ref.
Expressor	0.997 (0.57-1.82)	0.99	0.95 (0.52-1.74)	0.86	1.376 (0.75-2.49)	0.308	1.37 (0.75-2.51)	0.305
Donor CYP3A5 genotypes								
Nonexpressor	Ref.	Ref.	Ref.	Ref.	Ref.	Ref.	Ref.	Ref.
Expressor	1.46 (0,80-2.67)	0.21	1.33 (0.67-2.53)	0.87	1.87 (1.02-3.43)	0.043	2.04 (1.06-3.92)	0.032
GRWR								
≤3%	Ref.	Ref.	Ref.	Ref.	Ref.	Ref.	Ref.	Ref.
>3%	1.14 (0.63-2.08)	0.66	1.01 (0.53-1.91)	0.98	1.67 (0.91-3.04)	0.095	1.78 (0.94-3.38)	0.077

## Data Availability

The data used to support the findings of this study are available from the corresponding author upon request.

## Abbreviations

BMI: Body mass index
C/D: Concentration/dose
CSA: Cyclosporine
CYP: Cytochrome P450
D/R: Donor/recipient
EX: Expressor
GLMM: Generalized linear mixed model
GRWR: Graft-to-recipient body weight ratio
LDLT: Living donor liver transplantation
LS: Large-size
LLS: Left lateral segment
LT: Liver transplantation
MHV: Middle hepatic vein
MMF: Mycophenolate mofetil
NEX: Nonexpressor
PELD: Pediatric end-stage liver disease
PFIC: Progressive familial intrahepatic cholestasis
SS: Small-size
TAC: Tacrolimus.
